# Comparison of the Effect of Nd:YAG and Diode Lasers and Photodynamic Therapy on Microleakage of Class V Composite Resin Restorations

**DOI:** 10.5681/joddd.2013.013

**Published:** 2013-05-30

**Authors:** Siavash Savadi Oskoee, Parnian Alizadeh Oskoee, Elmira Jafari Navimipour, Amir Ahmad Ajami, Fatemeh Pournaghi Azar, Sahand Rikhtegaran, Melina Amini

**Affiliations:** ^1^Professor, Dental and Periodontal Research Center, Tabriz University of Medical Sciences, Tabriz, Iran; ^2^Associate Professor, Department of Operative Dentistry, Faculty of Dentistry, Tabriz University of Medical Sciences, Tabriz, Iran; ^3^Assistant Professor, Department of Operative Dentistry, Faculty of Dentistry, Tabriz University of Medical Sciences, Tabriz, Iran; ^4^Post-graduate Student, Department of Operative Dentistry, Faculty of Dentistry, Tabriz University of Medical Sciences, Tabriz, Iran

**Keywords:** Diode laser, microleakage, Nd:YAG laser, photodynamic therapy

## Abstract

**Background and aims:**

Considering the importance of disinfecting dentin after cavity preparation and the possible effect of disinfection methods on induction of various reactions between the tooth structure and the adhesive restorative material, the aim of the present study was to evaluate microleakage of composite resin restorations after disinfecting the prepared dentin surface with Nd:YAG and Diode lasers and photodynamic therapy.

**Materials and methods:**

Standard Class V cavities were prepared on buccal surfaces of 96 sound bovine teeth. The samples were randomly divided into 4 groups based on the disinfection method: Group 1: Nd:YAG laser; Group 2: Diode laser; Group 3: photodynamic therapy; and Group 4: the control. Self-etch bonding agent (Clearfil SE Bond) was applied and all the cavities were restored with composite resin (Z100). After thermocycling and immersing in 0.5% basic fuchsin, the samples were prepared for microleakage evaluation under a stereomicroscope. Data was analyzed with Kruskal-Wallis and Wilcoxon signed-rank tests at P<0.05.

**Results:**

There were no significant differences in the microleakage of occlusal and gingival margins between the study groups (P>0.05). There were no significant differences in microleakage between the occlusal and gingival margins in the Nd:YAG laser group (P>0.05). In the other groups, microleakage at gingival margins was significantly higher than that at the occlusal margins (P<0.05).

**Conclusion:**

Nd:YAG and Diode lasers and photodynamic therapy can be used to disinfect cavity preparations before composite resin restorations.

## Introduction

Recurrent caries is one of the most common problems after tooth restorative procedures.^1^ Many authors have attributed recurrent caries, pulp inflammation and necrosis to microleakage.^2^ Failure to remove infected tooth structures during cavity preparation aggravate problems associated with cavity margin microleakage. Bacteria remaining on the dentinal cavity floor can preserve their viability for a long time.^3^ Leung et al reported that the number of bacteria remaining in the cavity can double during a one-month period after the restorative procedure.^4^Therefore, removal of the infected dentin is important to prevent recurrent caries, and disinfection of dentin is recommended after cavity preparation.^5^ Several techniques have been introduced to this end. Different kinds of chemical agents, including sodium hypochlorite, chlorhexidine, EDTA (Ethylenediaminetetracetic Acid), hydrogen peroxide, povidone-iodine, citric acid, triclosan, glutaraldehyde, calcium hydroxide, silver nitrate, halogens, some lasers, ozone therapy equipment and photodynamic therapy have been evaluated in relation to their antimicrobial effects.^6-8^

A possible problem with chemical agents is their tissue toxicity at the concentrations used.^9^ In addition, an in vitro study has shown that a large number of bacteria are still viable even after application of povidone-iodine or sodium hypochlorite for 15 minutes.^10^

Application of lasers has been extensively studied in operative dentistry as an alternative for burs for cavity preparation, treatment of dentin hypersensitivity and preparation of dentin before application of adhesive systems. The efficacy of lasers has been shown in occluding and opening the dentinal tubules (depending on the energy level used), producing microscopic surface irregularities without demineralization, and sterilization of the dentin surface.^11,12^

Nd:YAG laser (Neodymium: Yttrium-Aluminum-Garnet) is a pulsed infrared laser with a wavelength of 1064 nm; it is highly absorbable in pigmented tissues.^13^ This laser could be used in tooth hard structures for increasing resistance to acid attacks, remineralization of incipient caries, debridement and alteration of enamel pits and fissures to prevent carious lesions, disinfection of cavity preparations,^13^ treatment of dentin hypersensitivity,^14^ apical seal of endodontic obturations, decreasing root canal bacterial counts,^15^ sterilization of laser-irradiated surfaces and increasing penetration of fluoride into the enamel.^16^ It may result in liquefaction and re-crystallization of laser-irradiated enamel and dentin surfaces, producing a glass-like morphologic appearance, which is a surface devoid of any microorganisms.^13^

Diode laser is a laser produced by stimulation of Gallium and Arsenide, with or without Aluminum or Indium. It has a wavelength of 800–1064 nm. Hemoglobin and pigmented tissues and materials are most affected by the 810–830-nm wavelength of this laser.^17^ Gutknecht et al reported that the Diode laser can eliminate bacteria up to a depth of 500 µm in dentin at a wavelength of 980 nm, compared to chemical agents which penetrate only to a depth of 100 µm.^18^

Photodynamic therapy is a treatment modality in which the chemicals used become activated and release reactive cytotoxic oxygen species at a certain wavelength. These chemicals have a penetrating capacity and can become active within the tissue, which is the basis for photodynamic therapy.^9^ At first, the target cells are selectively subjected to the sensitizer and then irradiated by a complementary wavelength.^19^ This technique has exhibited high efficacy in the treatment of neoplasms.^9^ Photo-activated disinfection or photodynamic antimicrobial therapy (PACT) is a term used for the disinfecting protocol in which bacterial cells are targeted instead of malignant cells.^9^

This technique is effective against a large number of gram-positive and gram-negative bacteria of the oral cavity with the use of different sensitizers and various wavelengths. Recent studies have shown that this technique can eliminate bacteria in planktonic culture media and samples taken from the plaque and biofilm.^19^ The success of this technique depends on factors such as bacterial sensitivity, the type of the photosensitizer used, the time needed for the delivery of photosensitizer and irradiation duration.^19^

All the lasers mentioned above would have different effects on tooth hard structures and interactions with the dentin; a study has shown that Nd:YAG laser is more effective in decreasing the diameter of dentinal tubules compared to the diode laser.^20^ Although previous studies have shown the negative effect of oxygen and other oxidating agents (bleaching agents) on the bond strength of adhesives, the effect of photodynamic therapy on the bonding process is still unknown. The aim of the present study was to evaluate microleakage of composite resin restorations at occlusal and gingival margins after the application of Nd:YAG and Diode lasers and photodynamic therapy.

## Materials and Methods

Ninety-six sound bovine incisors were used in the present in vitro study. The teeth were stored in 0.5% chloramine T solution before the study. All the teeth were sealed and cleaned by pumice and a rubber cup. Standard Class V cavities were prepared on the buccal surfaces,with the dimensions of 2 mm in depth, 2 mm in the mesiodistal and 3 mm in the occlusogingival dimensions;^22^ the occlusal and gingival margins were both placed 1.5 mm occlusal and apical to the CEJ, respectively. A sharp diamond fissure bur in a high-speed handpiece along with air and water spray was used for cavity preparation.^23^ A new bur was used for every 5 cavity preparations.^1^ The samples were randomly divided into 4 groups of 24 based on the preparation procedure as follows:

Group 1: Nd:YAG laser (Nd:YAG Dental Laser, Lambda Scientifica Srl, Vicenza, Italy); based on manufacturer’s instructions the parameters of the laser beam used were as follows: a pulsed wavelength of 1.064 µm; non-contact with a distance of 1 mm from the surface; an output power of 1.5 W; an energy level of 50 mJ; and a frequency of 15 Hz for 10 seconds. The fiber optic diameter was 400 µm.

Group 2: Diode laser (Chesse^TM^ 4W Mini Dental Diode Laser, Wuhan Gigaa Optronics Technology CO, Ltd, China); the parameters of the laser beam used were as follows: a wavelength of 810 nm; an output power of 1 W; and continuous mode. The fiber optic diameter was 200 µm.

Group 3: Photodynamic therapy: based on manufacturer’s instructions the tolunium chloride solution (Pharmaceutical grade of the vital stain, Toluidine blue O) was placed over samples with a concentration of 12.7 mg/L. Then the samples were irradiated with a Diode laser beam (RJ-LASER, Fabrikstr 2279183, Walkirch, Germany) at a wavelength of 655 nm and a power of 10 J/cm^2^ using the continuous mode; a dental bar head measuring 70 mm in length and 8 mm in diameter was used for 120 seconds.

Group 4: The control; the samples did not undergo any antimicrobial procedures.

Subsequent to the above-mentioned antimicrobial procedures, self-etch bonding agent (Clearfil SE Bond, Kuraray Co, Ltd, Osaka, Japan) was applied based on manufacturer’s instructions. The primer was applied for 20 seconds and was gently dried. The bonding agent was applied and light-cured for 10 seconds. The cavities in all the groups were restored using bulk technique with composite resin (Z100, 3M ESPE, Dental Products, St Paul, MN, USA) and light-cured using a halogen light-curing unit (Astralis 7, Ivoclar Vivadent, Schaan, Liechtenstein) at a light intensity of 400 mW/cm^2^ using a probe with a diameter of 8 mm, perpendicular and barely touching the surface for 40 seconds. The output of the light-curing unit was tested with a radiometer. Finally, the restorations were finished with diamond burs (Diamant Gmbh, D&Z, Berlin, Germany) and polished with polishing disks (Sof-Lex TM, 3M ESPE, Dental Products, St. Paul, USA).

The samples were stored in distilled water at 37°C for 48 hours and then underwent a thermocycling procedure consisting of 500 cycles at 5–55°C with a dwell time of 30 seconds and a transfer time of 10 seconds.^22^ Subsequently, the samples were covered with a layer of wax and all the tooth surfaces were covered with nail varnish expect for restoration surfaces and 1 mm around the margin of restorations. The samples were then placed in 0.5% fuchsin basic solution for 24 hours at room temperature.^1^ Finally, a diamond disk (Diamant Gmbh, D&Z, Berlin, Germany) was used in a low-speed handpiece under water spray to divide them into two halves at the center of the restoration. Microleakage was evaluated under a stereomicroscope (Nikon, SMZ 1000, Tokyo, Japan) at ×25.^21^

Dye penetration was evaluated at tooth–restoration interface based on the following criteria:

0: No dye penetration

1: Dye penetration at tooth–restoration interface up to the half of the cavity depth

2: Dye penetration to the whole cavity depth without the involvement of the axial wall

**Figure 1:**
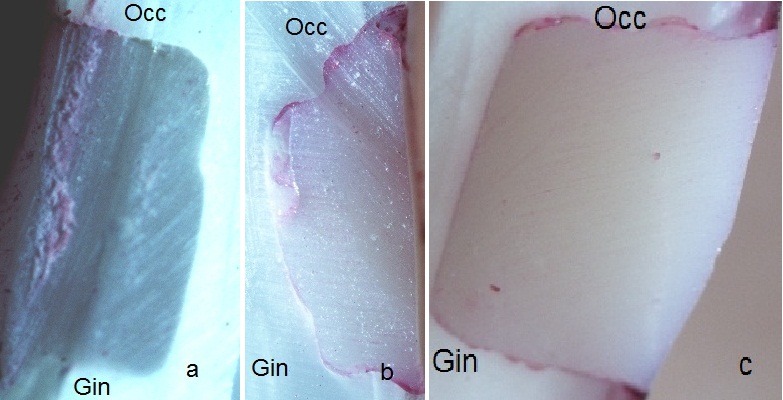
Scoring of microleakage based on dye penetration: (a) Score 0 at gingival margin and 1 at occlusal margin. (b) Score 2 at gingival and occlusal margin. (c) Score 3 at gingival and occlusal margin.

3: Dye penetration along the axial wall (Figure 1).

Non-parametric Kruskal-Wallis and Wilcoxon signed-rank tests were used to analyze data in relation to the amount of microleakage at occlusal and gingival margins at a significance level of P=0.05.

## Results

The descriptive results of microleakage at occlusal and gingival margins in the study groups are presented in Table 1. Kruskal-Wallis test did not reveal any significant differences in the occlusal and gingival margin microleakage between the study groups (P>0.05).

**Table 1:** Frequency of microleakage in the study groups

Groups	Margin	Microleakage scores
		0	1	2	3	(n)
1	Occlusal	4	12	8	0	24
	Gingival	4	12	8	0	24
2	Occlusal	3	16	5	0	24
	Gingival	1	10	13	0	24
3	Occlusal	9	11	4	0	24
	Gingival	3	15	6	0	24
4	Occlusal	9	12	3	0	24
	Gingival	1	13	10	0	24

Intra-group comparison of microleakage at occlusal and gingival margins by Wilcoxon signed-rank test did not reveal significant differences in group 1 (P=0.96).

In the other groups, microleakage at gingival margins was significantly higher than that at occlusal margins: P=0.004 in group 2; P=0.011 in group 3; and P=0.001 in group 4.

## Discussion

Microleakage is defined as the accumulation of bacterial fluids, molecules and ions between the cavity walls and the restorative materials, which is not clinically detectable^24^ and is one of the most important reasons for recurrent caries and pulpitis.^2^ There is evidence that it is not necessary to remove all the infected dentin adjacent to the pulp in order to control caries, and only elimination of soft and moist dentin and adequate obturation of the cavity with the restorative material is sufficient.^25^ Some studies have shown that dentinal bacteria can remain viable for a long time and can double in numbers one month after the restorative procedure.^4^ In addition, the majority of restorative materials available now do not

have the potential to obturate the cavity for a long time;^26^ therefore, it would be reasonable to disinfect dentin before restoration of the cavity in order to prevent recurrent caries.^5^ Some of these techniques include the use of various chemical agents and application of some laser types and photodynamic therapy. The most important factor for the efficacy of disinfection techniques is their potential for penetration. Bacteria can penetrate up to 1100 µm into the peri-luminar dentin,^27^ but disinfecting chemical agents can only penetrate up to 130 µm into the dentin.^28^ Lasers with a wavelength in the range of infrared waves, such as Nd:YAG and Diode lasers, have been used for various purposes, including removal of carious tooth structures, obturation of dentinal tubules and antibacterial activity.^29,30^ In this context, the bactericidal activity of Nd:YAG laser, which can penetrate more than 1000 µm into the dentin, has been used to eliminate bacteria from dentin.^31^ Penetration of lasers is due to the function of enamel rods and dentinal tubules as optic fibers.^32^ Gutknecht et al reported a 99.91% decrease in bacterial counts by Nd:YAG laser in the roots of extracted teeth.^33^

Widespread use of Diode laser has been reported in root canal treatment to overcome the problem of inadequate penetration of disinfecting agents, elimination of the smear layer produced due to instrumentation and its antimicrobial activity.^29^ Lee et al showed that laser irradiation through dentin disks measuring 500 µm eliminates 97.7% of Streptococcus mutans species, compared to a decrease of 54% in bacterial counts with the use of chlorhexidine, demonstrating a higher efficacy for the Diode laser.^34^

Wilson et al reported the use of photodynamic therapy as a technique to eliminate cariogenic bacteria and plaque-forming organisms in the presence of a photosensitizer and application of a low-power laser beam.^35^ Various other studies have used photosensitizer and different light sources other than that used by Wilson et al for their antimicrobial activity against various microorganisms.^36-39^ The technique has yielded good results in vivo and in vitro.^40-43^

Zanin et al used a light-emitting diode and toluidine blue on biofilms and reported a 95% decrease in Streptococcus mutans, S. sanguis and S. sorbinus counts.^39^

Interaction of laser with tooth hard structures is determined by radiation parameters, including wavelength, pulse energy, exposure duration, repetition rate and the optical properties of the tissue involved.^34^ The aim of the present study was to evaluate microleakage of composite resin restorations after the use of three disinfection techniques in Class V cavities. Based on the results the degree of microleakage in the study groups was not significantly different. Studies have yielded differing results in relation to the effect of Nd:YAG laser. Obeidi et al attributed a decrease in microleakage with the application of Nd:YAG laser to the energy of the laser beam.^2^ Kwaguchi et al^44^ reported that Nd:YAG laser had no effect on the marginal microleakage of composite resin restorations; however, Navarro et al^45^ reported a decrease in microleakage of composite resin restorations with the application of Nd:YAG laser, which is consistent with the results of a study carried out by White et al.^46^

In addition, Wen et al^47^ reported an increase in the tensile bond strength and a decrease in microleakage with the application of the laser. Dentin surface irradiation with Nd:YAG laser results in chemical and morphological changes. Chemical changes include an increase in calcium and phosphorus content and a decrease in oxygen concentration.^30^ Nd:YAG laser-irradiated dentin exhibits a glass-like surface due to heat liquefaction and re-solidification along with the liquefaction of the smear layer and the partial obturation of dentinal tubules and a decrease in microorganism counts.^13^

A decrease in dentin permeability results in a decrease in postoperative pain and an increase in resistance against solubility in acids during caries process. Elimination of the smear layer and microorganisms is in favor of the bonding process; however, liquefaction and obturation of dentinal tubules is not in favor of this process.^13^ The majority of studies on Diode laser have been carried out on root canals. Faria et al reported an increase in the apical microleakage with the application of Diode laser; they observed primary liquefaction and changes in the smear layer under an electron microscope.^48^ Costa Ribeiro et al^15^ and Esteves-Oliveira et al^49^ reported liquefaction and partial obturation of dentinal tubules after Diode laser irradiation.

Studies on photodynamic therapy have been predominantly carried out on microbial culture media and suspensions and there have been limited studies on dentin samples.^42,43,50^

In the present study, changes in the amount of microleakage were expected in the Nd:YAG and Diode laser groups due to the probability of dentinal surface changes and in the photodynamic therapy group due to the presence of photosensitizer agent (oxygen) and perhaps its interference with resin polymerization; however, it appears the lasers used with the parameters previously mentioned did not have the capacity to exert the expected dentinal surface changes and the amount of the oxygen in the photosensitizer agent was not sufficient to interfere with resin polymerization and increase microleakage. In the intra-group comparison of occlusal and gingival margins in the Diode laser, photodynamic therapy and control groups, microleakage at gingival margins was higher than that at occlusal margins, which might be attributed to polymerization shrinkage of composite resin, the forces of which have exceeded the bond strength to dentin, resulting in gaps at gingival margins. The effect of the factors above on obturation of dentinal tubules might be another reason involved. The nature of gingival margins, too, can be effective in this respect because dentin at gingival margins contains a significant amount of water, organic materials and a moist surface, which compromise the bonding mechanism and increase microleakage.^1^ However, in the Nd:YAG laser group there were no significant differences in microleakage between occlusal and gingival margins, which might be attributed to possible minor changes in gingival dentin by this type of laser and the decrease in microleakage at gingival margins.

Finally, it should be pointed out that although based on previous studies the above-mentioned techniques were used as cavity disinfecting agents, evaluation of dentin surface by electron microscopy, microbial culture and evaluation of thermal effects of the lasers on affected dentin were not carried out in the present study. In order to extend the results, it is suggested that future studies be carried out by implementing the conditions mentioned above.

## Conclusion

Under the limitations of the present study it can be concluded that Nd:YAG and Diode lasers and photodynamic therapy can be used for disinfection of cavities without any detrimental effect on microleakage.
